# Divergent Expression of CXCR5 and CCR5 on CD4^+^ T Cells and the Paradoxical Accumulation of T Follicular Helper Cells during HIV Infection

**DOI:** 10.3389/fimmu.2017.00495

**Published:** 2017-05-12

**Authors:** John Zaunders, Yin Xu, Stephen J. Kent, Kersten K. Koelsch, Anthony D. Kelleher

**Affiliations:** ^1^St Vincent’s Centre for Applied Medical Research, St Vincent’s Hospital, Sydney, NSW, Australia; ^2^The Kirby Institute, The University of New South Wales, Sydney, NSW, Australia; ^3^Department of Microbiology and Immunology, Peter Doherty Institute, University of Melbourne, Melbourne, VIC, Australia; ^4^Department of Infectious Diseases, Alfred Hospital, Monash University, Melbourne, VIC, Australia

**Keywords:** HIV, lymphoid tissue, CD4^+^ T lymphocytes, T follicular helper cells, germinal centers

## Abstract

Viral infection sets in motion a cascade of immune responses, including both CXCR5^+^CD4^+^ T follicular helper (Tfh) cells that regulate humoral immunity and CCR5^+^CD4^+^ T cells that mediate cell-mediated immunity. In peripheral blood mononuclear cells, the majority of memory CD4^+^ T cells appear to fall into either of these two lineages, CCR5^−^CXCR5^+^ or CCR5^+^CXCR5^−^. Very high titers of anti-HIV IgG antibodies are a hallmark of infection, strongly suggesting that there is significant HIV-specific CD4^+^ T cell help to HIV-specific B cells. We now know that characteristic increases in germinal centers (GC) in lymphoid tissue (LT) during SIV and HIV-1 infections are associated with an increase in CXCR5^+^PD-1^high^ Tfh, which expand to a large proportion of memory CD4^+^ T cells in LT, and are presumably specific for SIV or HIV epitopes. Macaque Tfh normally express very little CCR5, yet are infected by CCR5-using SIV, which may occur mainly through infection of a subset of PD-1^intermediate^CCR5^+^Bcl-6^+^ pre-Tfh cells. In contrast, in human LT, a subset of PD-1^high^ Tfh appears to express low levels of CCR5, as measured by flow cytometry, and this may also contribute to the high rate of infection of Tfh. Also, we have found, by assessing fine-needle biopsies of LT, that increases in Tfh and GC B cells in HIV infection are not completely normalized by antiretroviral therapy (ART), suggesting a possible long-lasting reservoir of infected Tfh. In contrast to the increase of CXCR5^+^ Tfh, there is no accumulation of proliferating CCR5^+^ CD4 T HIV Gag-specific cells in peripheral blood that make IFN-γ. Altogether, CXCR5^+^CCR5^−^ CD4 T cells that regulate humoral immunity are allowed greater freedom to operate and expand during HIV-1 infection, but at the same time can contain HIV DNA at levels at least as high as in other CD4 subsets. We argue that early ART including a CCR5 blocker may directly reduce the infected Tfh reservoir in LT and also interrupt cycles of antibody pressure driving virus mutation and additional GC responses to resulting neoantigens.

## Introduction

Primary HIV-1 infection invariably leads to life-long chronic infection characterized by viral replication, plasma viremia, and the slow decline of CD4^+^ T cell numbers (1). Within weeks of primary HIV-1 infection, patients have demonstrably high levels of both HIV-specific antibodies, which are routinely used to diagnose the infection (2), and HIV-specific CD8^+^ T cells (3).

This continued HIV-1 replication (in the absence of therapy) in the face of high levels of specific immune response contrasts with other typical acute viral infections in which clearance is associated with the emergence of neutralizing antibodies (nAb) and/or cytotoxic CD8^+^ T lymphocytes (CTL) (4). Since antibody responses and CD8^+^ T cells were believed to be mainly reliant on CD4^+^ T cells for help and these cells were the target of HIV-1 infection, there was widespread belief that, based primarily on peripheral blood assays, CD4^+^ T cell responses to HIV-1 were lost early and the antibody and CD8^+^ T cell responses were dysregulated and impaired (5).

However, we postulate that it is now clearer that the HIV-specific responses of T follicular helper (Tfh) CD4^+^ T cells, which are anatomically compartmentalized within the lymphoid tissue (LT) through their expression of CXCR5 and PD-1 and their lack of expression of CCR5, may play a central role in HIV-1 pathogenesis.

## Antibody Responses to HIV-1

**Figure 1:**
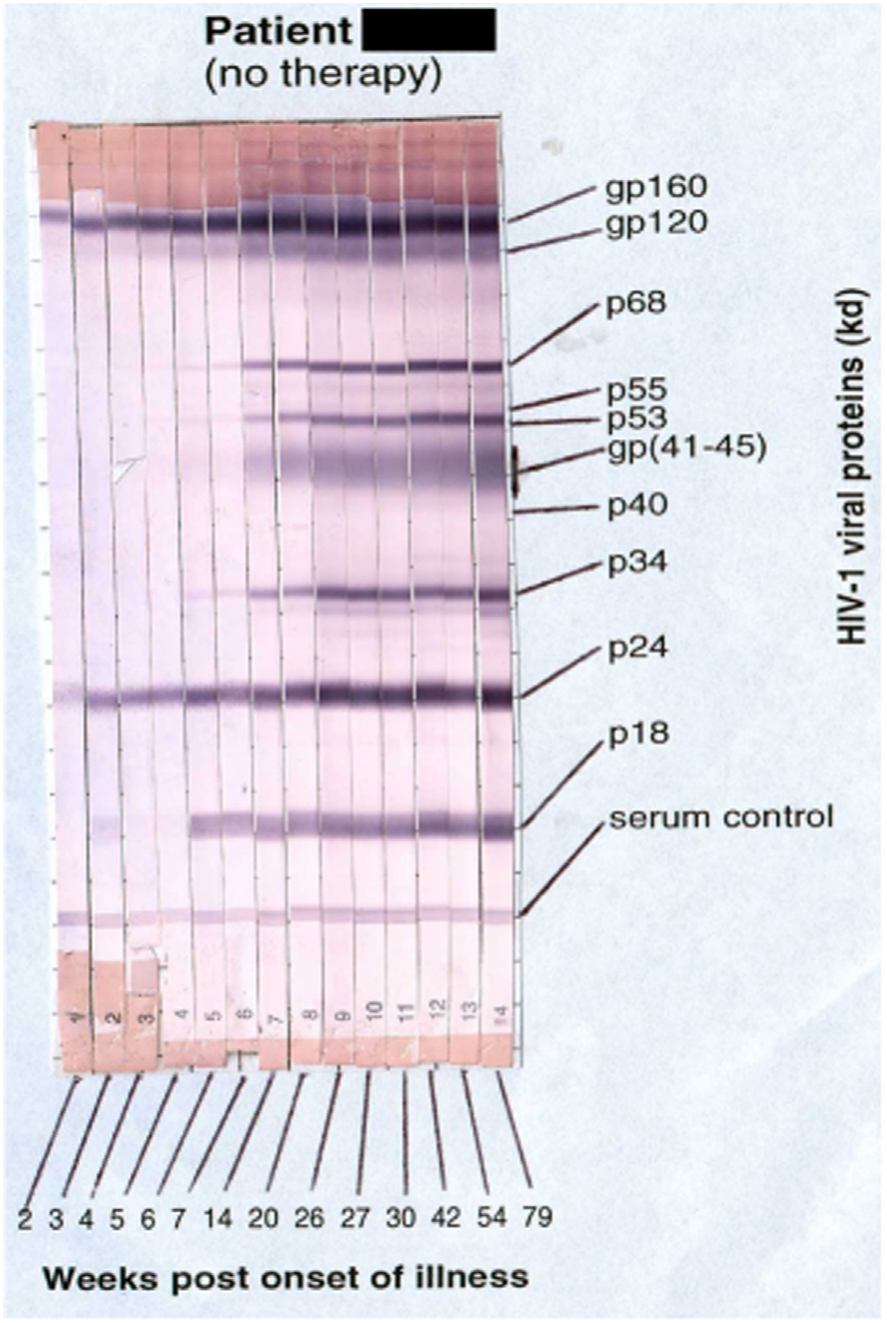
**Sequential appearance of anti-HIV antibodies in longitudinal serum samples from a subject with primary HIV-1 infection**. Shown are individual western blot strips for each sample, collected over 79 weeks following onset of symptoms (8) (photo courtesy of Philip Cunningham, St. Vincent’s Centre for Applied Medical Research, Sydney, NSW, Australia).

Primary HIV-1 infection usually leads to characteristic symptoms (2) a median of 21 days after exposure (6). Antibody responses to HIV-1 are first detectable by sensitive ELISA’s within about 1 week of onset of symptoms, and diagnostic western blots demonstrate a typical evolving increase in titer and breadth of the response to individual HIV-1 proteins over weeks to months (2, 7) (Figure 1).

Commencement of antiretroviral therapy (ART) very early during primary HIV-1 infection can actually lead to a decrease in anti-p24 titers (9) and overall antibody response (10) and to seroreversion on some diagnostic assays (11). Interruption of ART in such patients leads to a rapid rise in antibody levels, with faster increases than therapy-naïve patients (10).

Antiviral antibodies play an extremely important role in immunity to most viruses (4, 12), not just in preventing re-infection but also, in some cases, in helping to clear acute viral infection (13). Vaccine efficacy generally depends on antiviral nAb (4), but while HIV-1 is highly immunogenic, it was recognized very early that most anti-gp120 antibodies are non-neutralizing (14). Nevertheless, some HIV^+^ subjects with relative control over viral replication exhibit broadly directed neutralizing antibody activity (15) and antibody-mediated cell-mediated cytotoxicity of infected cells (16).

CD4^+^ T cells play a pivotal role in the development of effective humoral immunity to infections by helping B cells and also cellular immunity by helping CD8^+^ T cells, as well as in the regulation of the immune system. These varied helper functions are mediated through a range of subsets of CD4^+^ T cells, which have followed diverse cellular differentiation pathways (17).

In particular, Tfh cells, found in germinal centers (GC) during an immune response, are non-redundant in helping B cell responses to undergo somatic hypermutation and affinity maturation (18). The location of these cells within GC is dependent on their unique combined expression of the chemokine receptor CXCR5 and high levels of the checkpoint inhibitor PD-1, driven by the lineage-specific transcription factor, Bcl6 (18).

The kinetics and evolution of anti-HIV antibodies are highly suggestive of a role for HIV-specific CD4^+^ Tfh. However, using peripheral blood mononuclear cells (PBMC) and standard assays for assessing HIV-specific CD4 T cells, it was generally believed that only rare patients had significant CD4 responses to HIV proteins (19–24). LT was rarely sampled and Tfh were, until recently, not studied at all. It was widely assumed that a low or undetectable level of HIV-specific CD4 T cells in PBMC was consistent with the observations that HIV^+^ subjects rarely produced effective broadly nAb (7) when tested against standard HIV-1 strains.

Interestingly though, in the rhesus macaque model of primary SIV infection, it had originally been shown that CD8^+^ cell depletion prior to inoculation led to an inability to reduce the initial peak of viral load, which characteristically occurred by day 28 postinfection (25). This was widely interpreted as the essential role for CD8^+^ CTL to control viral replication (3). In contrast, B cell depletion prior to SIV infection did not affect the reduction in peak viral load, but after day 28, it was observed that viral load was inversely correlated with neutralizing antibody titer in the treated macaques, suggesting that humoral immunity did play a significant role in the control of established infection (26).

A turning point came when Richman et al. (27) showed that antibodies neutralized previous autologous envelope quasispecies, but that plasma virus in the same patients had continually and rapidly evolved to escape this neutralization. Similar results were found for changes in the glycan shield on the surface of gp120, again involving mutations in the sequence of *env* (28). These studies demonstrated two important points: (i) that nAb were actually applying significant pressure on viral replication in the individual patients, forcing viral escape as a result, and (ii) that new antibody responses were continually being generated.

A formal role for Tfh in maturation of anti-gp120 antibodies was confirmed by detailed studies showing very high levels of somatic mutations in B cells that produced broadly nAb (29). It has further been repeatedly demonstrated that most broadly nAb require high levels of somatic hypermutation (15). Altogether, these results imply a significant germinal center response to HIV-1 infection, which in turn implies a functional role for HIV-specific Tfh within them.

## The Massive Germinal Center Response in LT after Establishment of HIV-1 Infection

Histologic studies of lymph nodes have shown that follicular hyperplasia was characteristic of chronic HIV-1 infection. Hyperplastic lymph nodes were not seen immediately in primary HIV-1 infection, particularly in gut-associated LT (30). However, clinical diagnosis of peripheral lymphadenopathy was then frequently reported in untreated early, established infection (1). Furthermore, follicular hyperplasia that was present in lymph nodes prior to commencing ART was reduced in subsequent biopsies from the same individuals after 6 months of ART (31). Importantly, *in situ* hybridization has shown that the processes of follicular dendritic cells (FDC) within these GC retained a very large amount of HIV-1 virions attached to their processes [reviewed in Ref. (32)].

This follicular hyperplasia, observed in HIV infection, is often, but not always, replicated in the macaque model of SIV infection. One study counted the total number of GC in completely sectioned rhesus macaque lymph nodes finding that the average was ~200 GC/lymph node at day 270 postinfection, an eightfold rise from day 10 postinfection (33). An earlier study had reported that high SIV replication during primary SIV infection in rhesus macaques (RM) was generally associated with accumulation of high levels of virions on FDC cells within GC from 2 weeks post-inoculation (34). In contrast, in wild-caught sooty mangabeys with non-pathogenic natural SIV infection, lymph nodes showed normal histology and no evidence of virions trapped on FDC, despite high tissue viral loads (35). However, another study found that non-pathogenic infection of African green monkeys resulted in an elevation of germinal center B cell proliferation, with little proliferation in T cell areas of lymph nodes, compared to RM where there was less B cell area proliferation and more T cell area proliferation (36). It is possible that in some experimental infections of RM with highly pathogenic SIV, overwhelming lymph node infection may result in limited GC reactions, low antibody responses, and extremely rapid disease progression (34). Overall, though, GC are a prominent component of the response to SIV infection and may be related to the pathogenic course of experimental infection as a reservoir of virus.

Importantly, in HIV-1 infection, when the number of GC is combined with the number of lymph nodes and the number of attached virions, then the FDC-bound HIV-1 is calculated to represent the largest amount of virus in the body (32). In contrast, in the T cell areas of lymph nodes, relatively smaller numbers of HIV virions can be seen around relatively rare individual CD4 T cells, presumably the site of replicating HIV. These isolated CD4^+^ T cells are accompanied by large numbers of CD8^+^ T cells (32). Some studies suggest that CD8^+^ CTL do not enter GC, potentially providing a sanctuary site for HIV-infected CD4^+^ T cells (37, 38), although other studies have shown that CD8^+^ T cells with a cytotoxic phenotype are dramatically increased in lymph nodes (36, 39) and can be seen in follicles (34, 40, 41).

Entrapment of antigens on FDC is due to a general opsonization mechanism by specific antibodies interacting with CD21 on the FDC processes, designed to provide native antigens to B cells, to improve antibody affinity, *via* somatic hypermutation during the GC reaction. Therefore, one effect of the large amount of antibodies directed to HIV is to swamp GC with HIV proteins, making them the predominant foreign antigen within lymph nodes during early chronic infection.

Indeed, in the SIV study by Margolin et al. described above, 25% of the GC showed evidence that they were producing antibodies to gp120 (33). This indicates that on average, there were a remarkable 50 GC per lymph node in which the B cells were specific for SIV envelope.

## Tfh Cells During HIV-1 Infection

Many reports have suggested that the proportion of CD4 T cells is reduced in lymph nodes and particularly the lamina propria of gut-associated LT during primary SIV and HIV infections (42–45). However, other studies in the SIV model, where the absolute numbers of cells have been counted, reported that the total number of CD4 T cells in lymph nodes actually increases in early chronic infection (46–48), associated with an increased expression of the proliferation marker Ki-67. An increase of Ki-67^+^CD4^+^ T cells is also seen in HIV^+^ subjects both in LT (41) and in PBMC (49), particularly during primary HIV-1 infection (50).

As described above, the GC reaction during immune responses largely depends on the highly specialized Tfh lineage of CD4^+^ T cells, expressing the main Tfh transcriptional regulator, Bcl-6, and the characteristic production of IL-21. Phenotypically, Tfh are characterized by expression of the chemokine receptor CXCR5^+^, which directs these cells toward CXCL13 produced by stromal cells within B cell areas of LT (18). Tfh are also clearly characterized by very high expression of the negative co-stimulatory regulator, PD-1. Histological studies have shown that Tfh found in the center of GC have the highest expression of PD-1 (51). It has been suggested that PD-1 may be important in reducing motility of T cells, and the functional implications of high PD-1 expression are discussed below.

In contrast, Tfh were reported to lack expression of CCR5, also believed to be important in their unique localization compared to other CD4^+^ T cells (18). Therefore, we began with the hypothesis that Tfh in LT were driving the GC hyperreactivity and antibody response in HIV-1 infection, due to their lack of CCR5 expression, which would allow them to avoid HIV-1 entry. We and others found that indeed, there was a clear relative increase in Tfh compared to other CD4^+^ T cells in excision biopsies from both SIV and HIV-1 infections, respectively (51–55), as well as in serial fine-needle biopsies from SIV-infected macaques (56). However, the proportion of Tfh drops dramatically in SIV-infected macaques with AIDS (57), and, in small numbers of rapid progressor SIV-infected macaques, Tfh numbers may not accrue at all, associated with loss of differentiated memory B cells in spleen (58) and with reduced antibody responses (34, 58).

We recently accurately quantified the number of Tfh using ultrasound-guided fine-needle biopsies of inguinal lymph nodes in human volunteers, and found a significant fivefold increase in the proportion of CD4^+^ T cells that were Tfh, as well as a corresponding 10-fold increase in Tfh cell numbers, in treatment-naive HIV^+^ subjects, compared to healthy adult controls. There was an accompanying >100-fold increase in the number of germinal center B cells (CD38^high^Ki-67^+^CD20^high^Bcl6^+^) in treatment-naive HIV^+^ subjects, compared to healthy adult controls (59).

The question arises whether the expanded Tfh are specific for the SIV or HIV-1 proteins. It has proven difficult to demonstrate antigen specificity of non-human primate or human Tfh using standard assays *in vitro*, but some studies have reported IL-21-producing HIV-1 Gag- and Env-specific Tfh in lymph nodes and in circulating Tfh-like cells (53, 60). Conversely, it has been reported that the *in vitro* function of Tfh from HIV^+^ subjects was adversely affected by overexpression of PD-L1 on B cells (61), or by intrinsic differences in transcription factors (62). Another reason for the difficulty in detecting HIV-specific Tfh using intracellular cytokine assays is that it is likely that the amount of IL-21 secreted by Tfh needs to be extremely limited so that it is only acting on proximal cognate B cells within the GC (63). In contrast, a cytokine-independent assay, based on OX40 (CD134) upregulation by antigen-specific CD4^+^ T cells (64), was recently used to show a high level of HIV-1 Env-specific Tfh in immunized macaques (65). Given the large number of SIV gp120-specific GC, described above (33), it is highly likely, but remains to be conclusively shown, that the expanded Tfh are in large part SIV or HIV specific.

## SIV and HIV-1 Infection of Tfh Cells

Despite the expansion of Tfh, both as a proportion of CD4 T cells in LT as well as in absolute numbers, it was also found that Tfh were infected with SIV or HIV-1 DNA at a similar or higher frequency compared to other subsets of CD4^+^ T cells in LT (52, 54, 55, 62) and *in vitro* (55, 66). In the SIV model, this appears to be potentially productive infection, as spliced SIV RNA can be detected from purified Tfh cells (52), and this was recently also reported for Tfh in HIV^+^ controllers (67). *In situ* hybridization with increased sensitivity also showed that a significant fraction of productively infected cells can be seen within B cell follicles in both pathogenic SIV infection of RM (68, 69) and in human HIV infection (66, 68).

Therefore, there is a conundrum, with both infection and expansion of Tfh in the course of chronic SIV and HIV-1 infections.

## HIV-1 Co-Receptors on Tfh and PRE-Tfh

In addition to using cell surface CD4 for viral entry, HIV-1 and SIV also use chemokine receptors as co-receptors (70). CXCR4 was the first co-receptor discovered because of its use by lab-adapted HIV-1 *in vitro*, but the main co-receptor *in vivo* during transmission and early chronic infection is CCR5 for both HIV-1 and SIV (70). CXCR5 acting as a co-receptor for HIV-1 or SIV has been reported only rarely (71, 72). The question arises as to how Tfh are infected at such a high rate with CCR5-using SIV or HIV-1, if they do not express CCR5.

**Figure 2:**
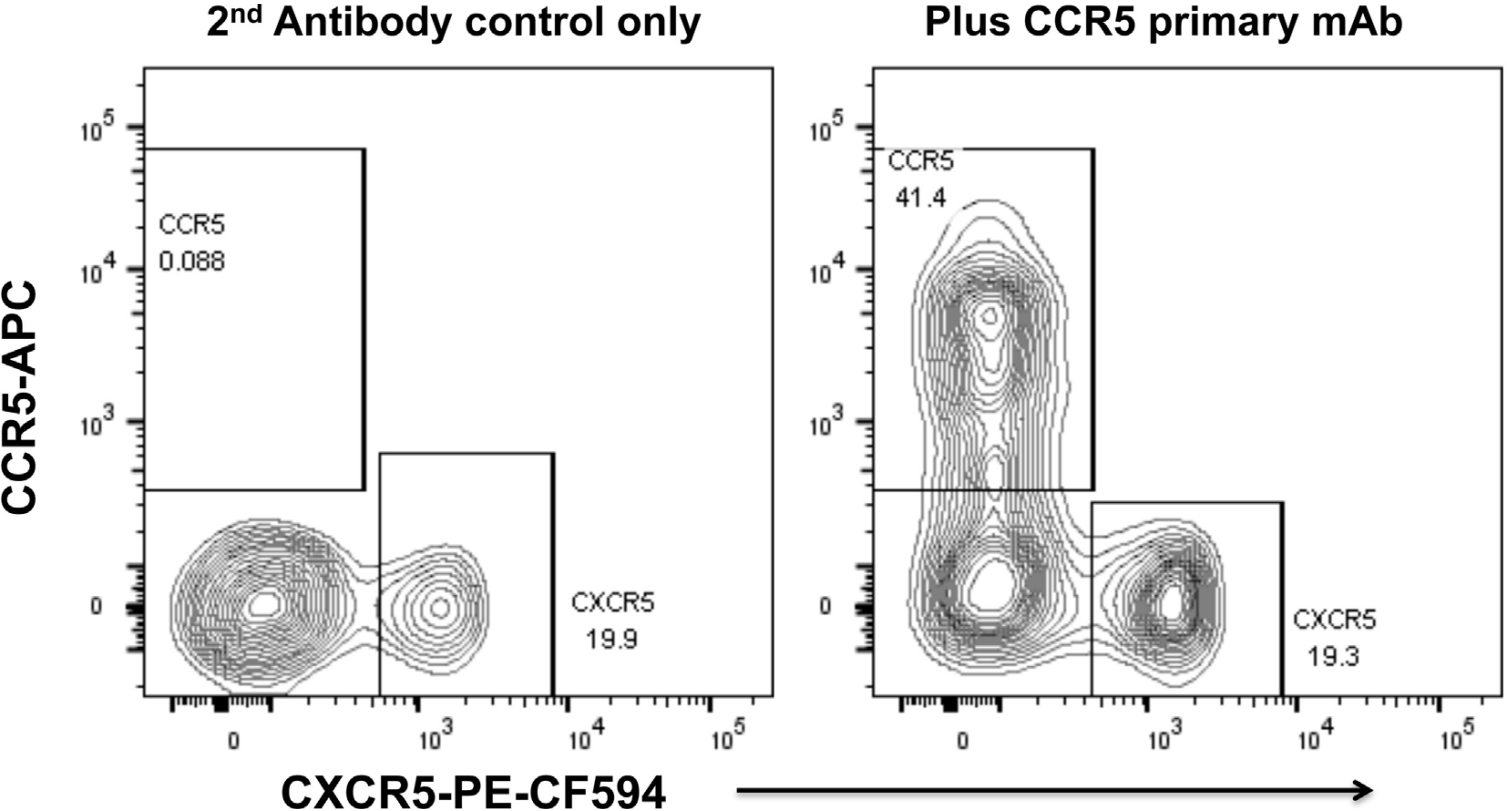
**Dichotomy of CCR5 and CXCR5 expression on memory CD4^+^ T cells in peripheral blood mononuclear cells, from a healthy adult control**. Representative flow plots show gated memory CD4^+^ T lymphocytes optimally stained for CCR5 (by indirect immunofluorescence) and CXCR5. Flowplot on left shows control staining without mAb for CCR5 (2° antibody only). Flowplot on right shows CCR5 staining versus CXCR5.

We have optimized staining for CCR5 on macaque and human lymph node cells (52, 73) and human PBMC (74), using indirect immunofluorescence staining. The results for human PBMC showed that there was a dichotomy of CCR5 and CXCR5 on memory CD4^+^ T cells in peripheral blood (74). Very few cells express both CCR5 and CXCR5, and surprisingly, double negative cells are also a minority (Figure 2), with a median of 29.9% of memory CD4 T cells being CCR5^−^CXCR5^−^ (74).

Similar results were also recently reported for rhesus macaque PBMC and lymph node cells (69).

When we studied LT from SIV-infected macaques, Tfh expressed very little, if any, CCR5, but the envelope sequences of amplified and cloned proviral SIV DNA from purified Tfh, were closely related to the original CCR5-using strains used to infect the pigtail macaques (52). The sequences phylogenetically intermingled with sequences from other subsets of memory CD4^+^ T cells, strongly suggesting that no specialized variant had arisen to specifically infect Tfh (52). Therefore, we hypothesized that Tfh infection was occurring *via* a precursor cell, a pre-Tfh cell, which was CCR5^+^ during the course of proliferation and differentiation following initial activation of the naïve CD4^+^ T cell.

In blood, during primary HIV-1 infection, the main population of proliferating CD4^+^ T cells are highly activated CD38^high^CCR5^+^Ki-67^+^ cells (50, 75), and include HIV-specific Th1 cells (75). However, these CD4^+^ T cells peak briefly at around 20 days after onset of symptoms (75), but thereafter do not accumulate despite maintaining an elevated rate of proliferation. This contrasts with the accumulation of activated proliferating CCR5^+^CD4^+^ T cells during acute EBV infection (50) and also after inoculation with Vaccinia virus (76, 77). Therefore, it was highly likely that activated CCR5^+^Ki-67^+^CD4^+^ T cells would be found in LT following HIV-1 infection.

In lymph node cells from SIV-infected macaques, we identified a population of PD-1^medium^CCR5^+^ memory CD4^+^ T cells (52) and found a high level of Ki-67 expression in these cells, whereas PD-1^high^ Tfh are largely Ki-67 negative (54). The high turnover of PD-1^medium^CCR5^+^ memory CD4^+^ T cells is consistent with a previous report of an increased proportion of Ki-67^+^ cells within CCR5^+^CD4^+^ T cells in peripheral blood from SIV-infected RM (78). A proportion of these PD-1^medium^CCR5^+^ memory CD4^+^ T cells also had a low level but unequivocal increase of expression of Bcl6 (73), as well as a proportion that clearly expressed ICOS (52). These results suggest that PD-1^medium^ memory CD4^+^ T cells may contain Bcl6^low^ICOS^+^ precursors of Tfh, but as described above, PD-1^medium^ memory CD4^+^ T cells also highly express CCR5, and we confirmed that they are highly infected with SIV DNA (52).

The possibility that there were putative pre-Tfh within the PD-1^medium^CCR5^+^ memory CD4^+^ T cell population is now supported by two recent reports (69, 73). The first reported that purified PD-1^neg/intermediate^CXCR5^−^CD4^+^ T cells from lymph nodes of uninfected macaques upregulated PD-1 and CXCR5 to high intensity after incubation with either anti-CD3/CD28/IL-4, plus IL-6, and IL-21 for 5 days *in vitro* (69), and the second reported that PD-1^intermediate^CD4^+^ T cells from lymph nodes incubated with anti-CD3/CD28 plus IL-21 upregulated both PD-1 and Bcl6 (73). At the same time as containing putative pre-Tfh cells, both studies found that PD-1^intermediate^CD4^+^ T cells from lymph nodes of SIV-infected macaques were infected at a high level with SIV DNA, and when differentiated to PD-1^high^ cells, also contained detectable SIV DNA (73). In particular, PD-1^intermediate^CD4^+^ T cells from lymph nodes include both CCR5^+^ and CCR5^−^ cells, and purified CCR5^+^PD-1^intermediate^CD4^+^ T cells can differentiate *in vitro* into PD-1^high^ cells (73). These results show that SIV infection of a CCR5^+^ pre-Tfh cell followed by final differentiation into a PD-1^high^ Tfh was plausible.

## Low Level Expression of CCR5 on Human Tonsillar Tfh

When we used optimized staining for CCR5 on Tfh from uninfected pigtail macaques, we observed very little, if any expression (52), but consistently observed a low level of expression of CCR5 on Tfh from SIV-infected macaques (52). Despite the original suggestion that Tfh do not express CCR5 (18), a recent report described that up to 30% of human Tfh obtained from human tonsil excisions from HIV-uninfected subjects were CCR5^+^ by flow cytometric analysis, with surprisingly similar or even higher percentages and mean fluorescence intensities compared to other lymph node CD4 subsets (66). Furthermore, it was shown in the same study that purified human tonsillar Tfh could be infected *in vitro* with CCR5-using HIV-1 (66). We have recently also found that median of 30–40% of human tonsillar Tfh are CCR5^+^, although they have a twofold to threefold lower mean fluorescence intensity for CCR5 than other CD4 (73). Furthermore, we also found that CCR5-using HIV-1 could not infect the majority CCR5-negative tonsillar Tfh, but could infect CCR5^+^ Tfh (73). Previously, it had been shown that human lymph node Tfh were highly permissive for *in vitro* infection with CXCR4-using lab strain NL4-3 (55).

It should be noted that Tfh in tonsils comprise a much higher proportion of CD4 than Tfh in unenlarged inguinal lymph nodes from healthy adults by fine needle biopsy (59), in spleen (62), or in gut biopsies (79), and therefore, tonsil may not be completely representative of all peripheral LT. Nevertheless, it appears that at least a subset of Tfh may be directly infected *in vivo* by CCR5-using HIV-1 early in infection. Tfh may also be relatively more permissive to direct infection because of their low level of the restriction factor, SAMHD1 (80).

## Dysregulation of GC

Normal immune responses are strictly controlled, by a large array of regulatory mechanisms including, but not restricted, to the dose of antigen (81), signaling through the check point inhibitor PD-1 (82), and suppressive activity of T regulatory cells (Tregs) (83). In the case of HIV-1 infection, as described above, virions are chronically retained in very large amounts in GC without clearance. Also, Tregs are likely targets for HIV-1 infection themselves, although there is no clear-cut deficit in their function in peripheral blood (84, 85). In our analysis of fine-needle biopsies, Tregs in inguinal lymph nodes showed no significant difference in number between HIV^+^ subjects and healthy controls (59).

A specific subset of CD4^+^ T cells, T follicular regulatory (Tfr) cells, have been described as Bcl6^+^Foxp3^+^ in mice and are present in very small numbers within GC, specifically to downregulate GC reactions (86–88). Although Tfr have not been definitively described in human LT, one study recently reported an increase of Foxp3^+^ cells within GC in lymph nodes from HIV^+^ subjects compared to controls, and there was a parallel increase in suppressive PD-1^high^CD25^+^Foxp3^+^ cells that formed a subset of the total increased PD-1^high^CXCR5^+^ Tfh in these tissues (89). However, the chronic increase in GC activity suggests that any possible increase in Tfr during HIV-1 infection is nevertheless overridden by the impetus to form GC.

It is only in end-stage HIV-1 infection that characteristic involuted GC are observed, interpreted as “burnt out” follicles (90), at a time when CD4 T cells in lymph nodes are almost completely depleted. Another important progressive effect on lymph node architecture during chronic HIV-1 infection is increasing deposition of collagen in T cell areas, which may severely and irreversibly disrupt CD4 T cell homeostasis (91).

## Expanded HIV-1 DNA-Infected Tfh as a Barrier to a Cure

In our experience, Tfh represent a median 3% of memory CD4^+^ T cells in inguinal lymph nodes in HIV^+^ subjects on ART (59), and a median of 4–5% in gut biopsies (79), although with a large range of observed results. Therefore, the number of HIV DNA-infected Tfh in the body, particularly when including the spleen (58, 62), will be very significant. Once the Tfh reservoir of HIV-1 DNA-infected cells is established, the question arises of what is their lifespan and how much do they contribute to the long-term reservoir, particularly for viral recrudescence if ART is interrupted. In fact, a recent study reported that the high frequency of HIV-1 DNA infection of Tfh persists following ART (92), but any relationship of this reservoir to rebounding plasma viremia is currently unknown.

Since Tfh lack IL-7R, they occupy a different cytokine-maintained niche to central memory CD4^+^ T cells or IL-2R^+^ Tregs. What keeps them alive, in either the short- or long-term, remains unclear. There was no evidence that expanded Tfh in lymph nodes from SIV-infected macaques had differentially high expression of IL-21R (52), but expanded human Tfh from HIV^+^ subjects do express high levels of ICOS (59), and ICOSL expression on B cells is known to be essential for full Tfh maturation and maintenance [reviewed in Ref. (93)].

Recent studies of fate mapping of Tfh in mouse GC suggest that when GC’s collapse, a very small subset of Tfh become resident cells in the outer follicle (94), under normal circumstances. Whether this occurs in HIV-1 infection, where follicular hyperplasia is long lasting, even in some patients on ART (59, 95), is unknown.

Studies of PBMC have shown that PD-1^+^ memory CD4^+^ T cells have a higher rate of HIV-1 DNA infection than other subsets (96). Further, the level of PD-1 expression in PBMC was correlated with a rapid recrudescence of plasma viremia during ART interruption (97). Since PD-1 ligation on human T cells negatively regulates T cell activation *via* TCR signaling (98), it is tempting to speculate that high levels of PD-1 on Tfh maintain HIV-1 DNA-infected Tfh in a state of latency. Against this, however, as discussed above, we and others did find evidence of productive infection of Tfh *in vivo* (52, 66, 68, 69).

Another open question is why it is that strategies to reduce T cell activation offer little or no benefit as adjuncts to ART, e.g., cyclosporine A (99), corticosteroids (100), mycophenylate motefil (101), chloroquine (102), or even recombinant IL-2 (103), which is known to antagonize the generation of Tfh (104).

## Conclusion

Increased GC activity in early chronic HIV-1 infection has largely been regarded as an epiphenomenon, and the high titers of anti-HIV antibodies have a limited role in control of viral replication or pathogenesis. However, the relatively recent re-emergence of interest in nAb, that require high levels of somatic hypermutation, has shifted the focus of much research back to GC.

**Figure 3:**
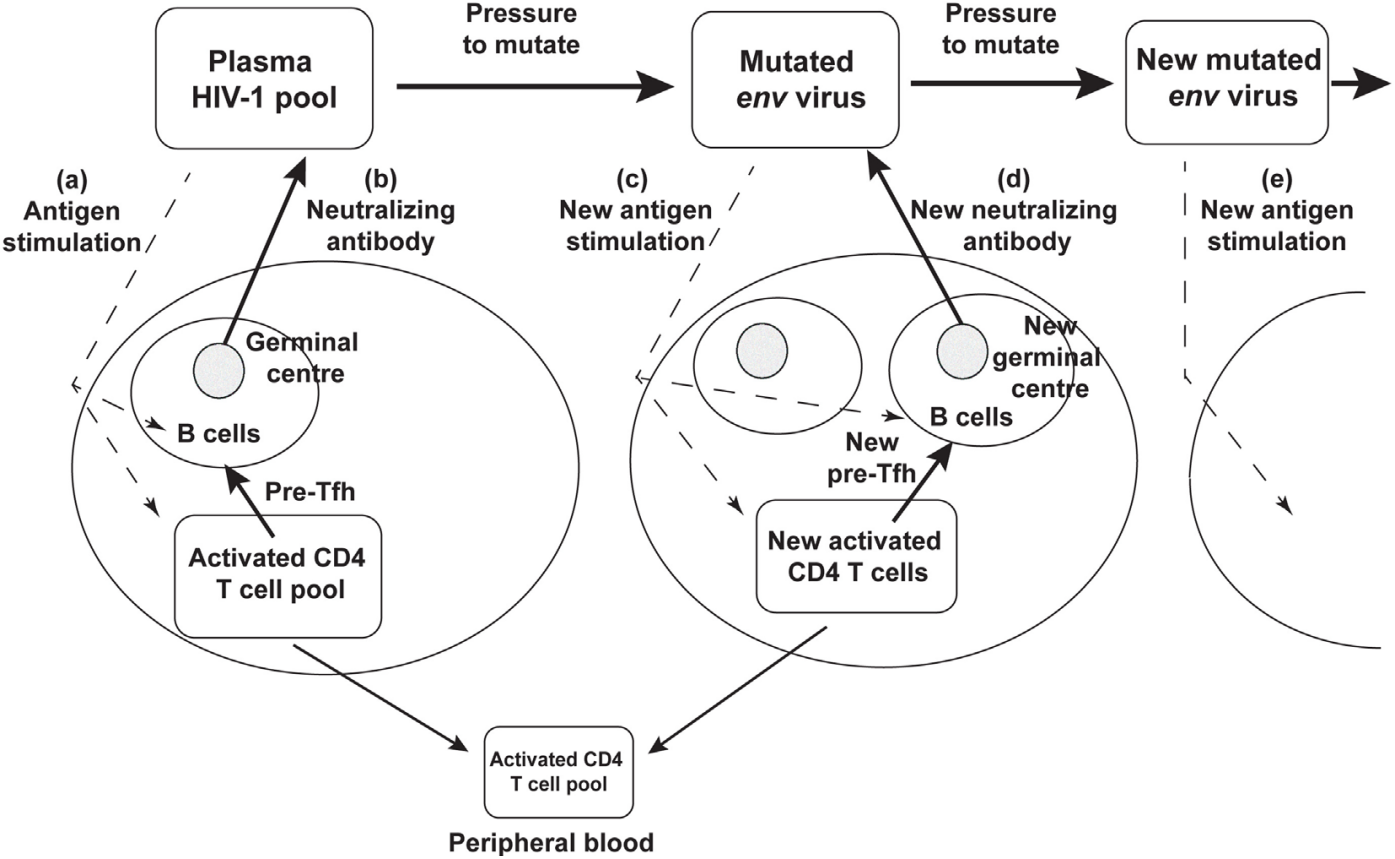
**Schematic diagram of neutralizing antibody pressure leading to new antibody responses to new epitopes, and an increasing number of germinal centers (GC), over time, driven by escape mutant viral quasispecies**. **(a)** HIV-1 stimulates T follicular helper (Tfh) and B cells to form a GC, and **(b)** produce neutralizing antibody. The neutralizing antibody then drives new env mutations that create neoantigens that **(c)** stimulate a new Tfh and B cells and GC reaction, that **(d)** produces a new neutralizing antibody, that drives further mutation and **(e)** further neoantigens for further Tfh and B cell responses.

Rather than an impotent immune response to HIV-1 infection, it now seems likely that the success of the humoral immune response drives continued *env* divergence and diversification (27, 28, 105). This in turn drives viral neoantigens which in turn may contribute to the slow recruitment of naïve CD4 T cells to the response, to chronic generation of activated target CCR5^+^ CD4 T cells, to further viral replication and thus significantly to the eventual depletion of CD4 T cells, as shown schematically in Figure 3.

Overall, it appears that the CCR5^−^ humoral immune lineage of CD4^+^ T cells provides the majority of the precursors to the expansion of Tfh cells in parallel to the viral antigen evolution as described above. However, there also appear to be CCR5^+^ precursors of Tfh as well as the low level expression of CCR5 on some Tfh themselves, and together these CCR5^+^ cells contribute to the pool of infected Tfh.

Therefore, even though the distinct patterns of expression of the two chemokine receptors CCR5 and CXCR5 align with the cellular versus humoral arms of the CD4^+^ T cell immune response, there appears to be some limited cellular overlap of expression during differentiation which accounts for the observed paradoxical combination of simultaneous accumulation and infection of Tfh during SIV and HIV-1 chronic infections.

CCR5 blockade is a logical approach to help prevent Tfh infection, possibly interrupting the chronic infection, based on our *in vitro* results. However CCR5 antagonists developed so far are generally not particularly potent drugs *in vivo*, and there is only one that has been licensed, maraviroc, which is not generally recommended for first-line ART (106). Intensification of ART with maraviroc in two studies have shown a trend to a decrease in HIV DNA levels in PBMC over 12–48 weeks (107, 108), but its effect on lymph node reservoirs was not investigated, although tissue penetration of maraviroc *in vivo* should have been adequate (109). Improved means to block entry *via* CCR5, including gene therapy approaches (110) would possibly result in a stronger reduction of the important Tfh reservoir.

## Author Contributions

All authors listed have made substantial, direct, and intellectual contribution to the work and approved it for publication.

## Conflict of Interest Statement

The authors declare that the research was conducted in the absence of any commercial or financial relationships that could be construed as a potential conflict of interest.
